# Omega-3 fatty acid intake during pregnancy and risk of infant maltreatment: a nationwide birth cohort – the Japan Environment and Children's Study

**DOI:** 10.1017/S0033291721002427

**Published:** 2023-02

**Authors:** Kenta Matsumura, Kei Hamazaki, Akiko Tsuchida, Hidekuni Inadera, the Japan Environment and Children's Study (JECS) Group

**Affiliations:** 1 Toyama Regional Center for Japan Environment and Children's Study, University of Toyama, Toyama, Japan; 2 Department of Public Health, Faculty of Medicine, University of Toyama, Toyama, Japan; 3 Department of Public Health, Graduate School of Medicine, Gunma University, Gunma, Japan

**Keywords:** Child abuse, child maltreatment, infant abuse and neglect, n-3 polyunsaturated fatty acids, seafood

## Abstract

**Background:**

Intake of omega-3 polyunsaturated fatty acids (PUFAs) has favorable effects, including reducing violent and aggressive behaviors, but its association with infant maltreatment is unknown. We therefore tested the hypothesis that maternal intake of omega-3 PUFAs is associated with a lower risk of infant maltreatment.

**Methods:**

Participants were 92 191 mothers involved in the ongoing Japan Environment and Children's Study. Omega-3 PUFA intake during pregnancy was measured using a food frequency questionnaire. Infant maltreatment was assessed using a self-reported questionnaire administered at 1 and 6 months postpartum.

**Results:**

Analysis using the lowest quintile of intake as a reference revealed that the adjusted odds ratios (ORs) with 95% confidence intervals (CIs) for cases of ‘hitting’ decreased as quintiles increased, with values for the second to fifth quintiles of 0.93 (95% CI 0.77–1.13), 0.79 (95% CI 0.64–0.97), 0.78 (95% CI 0.64–0.96), and 0.72 (95% CI 0.59–0.89), respectively. Adjusted ORs (95% CIs) for ‘shaking very hard’ at 6 months were 0.87 (0.73–1.04), 0.81 (0.67–0.97), 0.73 (0.61–0.89), and 0.78 (0.65–0.94), respectively. Adjusted ORs for ‘leaving alone at home’ for the second to fifth quintiles were 0.92 (0.87–0.98), 0.91 (0.86–0.97), 0.94 (0.88–0.99), and 0.85 (0.80–0.90), respectively.

**Conclusions:**

Higher maternal intake of omega-3 PUFAs during pregnancy was associated with fewer cases of hitting and violent shaking and leaving the child alone at home, implying a lower risk of infant maltreatment. Our results indicate the potential applicability of omega-3 PUFAs in reducing infant maltreatment.

## Introduction

Child maltreatment is the abuse or neglect of children aged 0–17 years old that leads to potential or actual harm to them (Krug, Dahlberg, Mercy, Zwi, & Lozano, 2002; World Health Organization, 2006). Although the prevalence of child maltreatment shows considerable heterogeneity according to country, type of maltreatment, and children's age (Finkelhor, Turner, Shattuck, & Hamby, 2013; Hillis, Mercy, Amobi, & Kress, 2016; Moody, Cannings-John, Hood, Kemp, & Robling, 2018), a 2017 UNICEF report (United Nations Children's Fund, 2017) states that 300 million (3 in 4) young children worldwide are regularly subjected to maltreatment by their caregivers. The most severe consequences of child maltreatment include injury, serious sequelae, and even death (Chevignard & Lind, 2014; Palusci & Covington, 2014). Most such deaths occur in infants (Palusci & Covington, 2014). The lifetime consequences include depression, smoking, obesity, high-risk sexual behavior, substance use, perpetration, and suicide attempt (Hughes et al., 2017; Thepthien & Htike, 2020), which in turn can lead to some of the principal causes of death, disease, and disability, including cardiovascular disease, sexually transmitted diseases, cancer, and suicide (World Health Organization, 2006). Therefore, public health measures must address prevention and intervention for child maltreatment.

Various psychopathological, socio-economical, and environmental factors are known to contribute to child maltreatment by mothers (Clement, Berube, & Chamberland, 2016; Palusci, 2011; Stith et al., 2009; Wu et al., 2004), but addressing many of these factors through intervention can be difficult. One potentially protective factor that could easily be targeted in intervention, but has currently received little attention, is maternal intake of omega-3 polyunsaturated fatty acid (PUFA). Omega-3 PUFAs are essential fatty acids involved in a wide range of vital activities and include docosahexaenoic acid (DHA) and eicosapentaenoic acid (EPA), both of which are found in fish oil. Meta-analyses and literature reviews have shown that omega-3 PUFA intake is effective for reducing violent and aggressive behaviors (Appleton, Rogers, & Ness, 2008; Gajos & Beaver, 2016; Hamazaki & Hamazaki, 2008). In addition, animal studies have revealed that omega-3 rich feedings promote nurturing maternal behavior (Asch, Schurdak, & McNamara, 2019; Harauma, Sagisaka, Horii, Watanabe, & Moriguchi, 2016). For example, homicide mortality rates are inversely related to seafood consumption in a country-specific manner (Hibbeln, 2001). Furthermore, interpersonal aggression is suppressed by DHA and EPA supplementation (Hamazaki et al., 1996), especially in stressful situations or in individuals under stress (Appleton et al., 2008; Hamazaki & Hamazaki, 2008). Dams fed a diet rich in omega-3 PUFAs show less infanticidal behavior (Harauma et al., 2016). However, to our knowledge, no previous studies have tested these favorable effects of PUFAs in the context of child maltreatment.

In this study, we used data obtained from an ongoing nationwide birth cohort study in Japan to examine the association between maternal intake of omega-3 PUFAs during pregnancy and risk of infant maltreatment. We hypothesized that mothers with a higher intake of omega-3 PUFAs would exhibit less infant maltreatment.

## Methods

### Study design and population

**Fig. 1.**
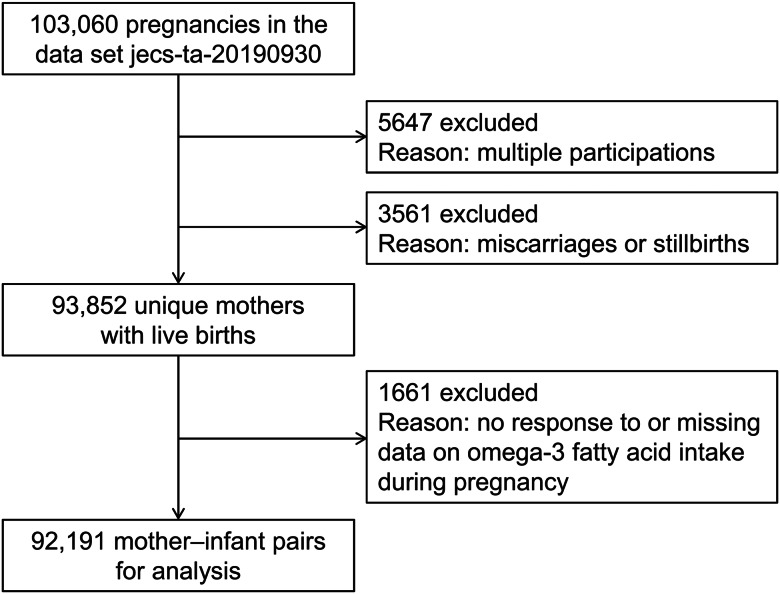
Participant flow chart.

Participants were members of the Japan Environment and Children's Study (JECS). The JECS is an ongoing, nationwide, government-funded birth cohort study focusing on various environmental factors and child health and development. The design of the JECS has been reported in detail elsewhere (Kawamoto et al., 2014; Michikawa et al., 2018). Briefly, expectant mothers were enrolled from 15 regional centers (including both rural and urban locations) in Japan via face-to-face recruitment between January 2011 and March 2014. Follow-up was conducted during the second or third trimester, at childbirth, and at 1 month postpartum during scheduled in-hospital checkups. Subsequent follow-ups were conducted at 6 months postpartum by mail. The present study analyzed the jecs-ta-20190930 dataset, which was released in October 2019. This dataset includes data on 103 060 pregnancies. Of these, 5647 multiple participations and 3561 miscarriages/still births were excluded to derive unique mothers. Among the remaining 93 852 mothers, 1661 were further excluded due to lack of responses or missing data on omega-3 PUFA intake during pregnancy. Finally, a total of 92 191 mother–infant pairs were analyzed (Fig. 1).

The authors assert that all procedures contributing to this work comply with the ethical standards of the relevant national and institutional committees on human experimentation and with the Helsinki Declaration of 1975, as revised in 2008. The JECS protocol was reviewed and approved by the Ministry of the Environment's Institutional Review Board on Epidemiological Studies (100910001) and the ethics committees of all participating institutions. Written informed consent was obtained from all participants. This specific study was also approved by the Ethics Committee of University of Toyama (R2020163).

### Measures

#### Exposure

Omega-3 PUFA intake during pregnancy (i.e. from the time of learning of pregnancy to the second or third trimester) was measured using a food frequency questionnaire (FFQ). The FFQ is a semi-quantitative instrument that assesses the average consumption of 171 food and beverage items, including 21 items related to fish or shellfish. The FFQ has been validated for use in large-scale Japanese epidemiologic studies (Sasaki, Kobayashi, & Tsugane, 2003; Yokoyama et al., 2016). To calculate the energy-adjusted intake using the residual model (Willett, Howe, & Kushi, 1997), we performed log-transformation of omega-3 PUFAs. Because 345 participants reported a value of 0 g/day for omega-3 PUFA intake, we replaced this value with 0.001 g/day, which is one-tenth of the lowest omega-3 PUFA intake of all participants (excluding 0 g/day). Energy-adjusted omega-3 PUFA intake was categorized into quintiles (Hamazaki et al., 2020) and then used as an exposure variable.

#### Outcome

Infant maltreatment was assessed via a self-reported questionnaire administered at 1 and 6 months postpartum. Because there is no clear gold standard as to what constitutes maltreatment (Moody et al., 2018), we carefully selected items involved in maltreatment by referring to the original definition (World Health Organization, 2006) and the definitions used in earlier surveys (Hussey, Chang, & Kotch, 2006; Straus, Hamby, Finkelhor, Moore, & Runyan, 1998) while also taking into consideration the sensitive nature of the subject matter. Items regarding maltreatment used in this study were as follows:

**Physical abuse**

- Hitting the baby (at 1 month postpartum)
- Shaking the baby very hard when he/she cries (at 1 month postpartum)
- Shook the child very hard in the past month (at 6 months postpartum)

**Neglect**

- Leaving the baby alone at home (at 1 month postpartum)

Mothers were instructed to indicate the frequency of these behaviors on a four-point Likert scale. Following the original definition of maltreatment (i.e. any behavior resulting in potential harm to the child; Krug et al., 2002; World Health Organization, 2006), answers to the aforementioned items other than ‘never’ were considered to indicate a case of maltreatment and were used as an outcome variable in this study.

#### Potential confounders

Based on existing evidence, we selected potential confounders as variables likely to affect both the prevalence of maltreatment (Fujiwara, Yamaoka, & Morisaki, 2016; Palusci, 2011; Stith et al., 2009; Wu et al., 2004) and the intake amount or physiological (functional) effectiveness of omega-3 PUFAs (de Groot, Emmett, & Meyer, 2019; Itomura et al., 2008; Lin, Huang, & Su, 2010; Mozaffarian, Bryson, Lemaitre, Burke, & Siscovick, 2005; Schiepers, de Groot, Jolles, & van Boxtel, 2010; Thesing, Bot, Milaneschi, Giltay, & Penninx, 2018). These variables included maternal age, pre-pregnancy body mass index, highest education level, full-time work, annual household income, smoking status, alcohol intake, parity, marital status, living with mother's parents, living with partner's parents, stressful events, intimate partner violence, negative attitude toward pregnancy, history of depression, anxiety disorder, dysautonomia, or schizophrenia, and psychological distress measured using the Japanese version (Furukawa et al., 2008; Sakurai, Nishi, Kondo, Yanagida, & Kawakami, 2011) of the Kessler Psychological Distress Scale (K6: Kessler et al., 2002). All the variables were categorized according to standard medical practice and common practice in Japan (Hamazaki et al., 2018; Matsumura, Hamazaki, Tsuchida, Kasamatsu, & Inadera, 2019). The categorizations are shown in Table 1.

**Table 1.** Participant characteristics according to quintile for energy-adjusted omega-3 polyunsaturated fatty acid (PUFA) intake during pregnancy

Variable	Quintile for energy-adjusted omega-3 PUFA intake during pregnancy										
	1 (low)		2		3		4		5 (high)		
	(*n* = 18 438)		(*n* = 18 439)		(*n* = 18 438)		(*n* = 18 438)		(*n* = 18 438)		
	*n*	(%)	*n*	(%)	*n*	(%)	*n*	(%)	*n*	(%)	*p*
Median intake, g/day	0.96		1.30		1.55		1.82		2.31		
Maternal age, y											<0.001
<25	2379	(12.9)	2019	(11.0)	1773	(9.6)	1669	(9.1)	1855	(10.1)	
25 to <30	5364	(29.1)	5273	(28.6)	5204	(28.2)	5137	(27.9)	5133	(27.8)	
30 to <35	6137	(33.3)	6446	(35.0)	6641	(36.0)	6627	(35.9)	6527	(35.4)	
⩾35	4558	(24.7)	4701	(25.5)	4820	(26.1)	5006	(27.2)	4923	(26.7)	
Pre-pregnancy body mass index, kg/m^2^											<0.001
<18.5	2899	(15.7)	2920	(15.8)	2931	(15.9)	3015	(16.4)	3185	(17.3)	
18.5 to <25	13421	(72.8)	13499	(73.2)	13662	(74.1)	13619	(73.9)	13327	(72.3)	
⩾25	2118	(11.5)	2021	(11.0)	1845	(10.0)	1804	(9.8)	1926	(10.5)	
Highest education level, y											<0.001
⩽12	7429	(40.3)	6775	(36.7)	6313	(34.2)	6207	(33.7)	6646	(36.0)	
>12 to <16	7592	(41.2)	7660	(41.5)	7910	(42.9)	7877	(42.7)	7740	(42.0)	
⩾16	3417	(18.5)	4004	(21.7)	4215	(22.9)	4355	(23.6)	4052	(22.0)	
Full-time work											<0.001
No	12257	(66.5)	12541	(68.0)	12514	(67.9)	13040	(70.7)	13455	(73.0)	
Yes	6181	(33.5)	5898	(32.0)	5924	(32.1)	5398	(29.3)	4983	(27.0)	
Annual household income, million JPY											<0.001
<4	8088	(43.9)	7545	(40.9)	7224	(39.2)	7182	(39.0)	7401	(40.1)	
4 to <6	5717	(31.0)	6067	(32.9)	6086	(33.0)	6192	(33.6)	6224	(33.8)	
⩾6	4633	(25.1)	4828	(26.2)	5128	(27.8)	5064	(27.5)	4813	(26.1)	
Smoking status											<0.001
Never	10100	(54.8)	10627	(57.6)	10934	(59.3)	10938	(59.3)	10664	(57.8)	
Former	7281	(39.5)	6930	(37.6)	6721	(36.5)	6798	(36.9)	6990	(37.9)	
Current	1057	(5.7)	882	(4.8)	784	(4.3)	702	(3.8)	785	(4.3)	
Alcohol intake											0.507
Never	6177	(33.5)	6125	(33.2)	6252	(33.9)	6084	(33.0)	6182	(33.5)	
Former	11736	(63.7)	11789	(63.9)	11666	(63.3)	11870	(64.4)	11762	(63.8)	
Current	525	(2.9)	525	(2.9)	521	(2.8)	484	(2.6)	495	(2.7)	
Parity											<0.001
Primipara	8226	(44.6)	7849	(42.6)	7795	(42.3)	7716	(41.9)	8011	(43.5)	
Multipara	10212	(55.4)	10590	(57.4)	10643	(57.7)	10722	(58.2)	10428	(56.6)	
Marital status											<0.001
Married	17299	(93.8)	17546	(95.2)	17685	(95.9)	17708	(96.0)	17645	(95.7)	
Single	886	(4.8)	751	(4.1)	618	(3.4)	572	(3.1)	639	(3.5)	
Divorced or widowed	254	(1.4)	142	(0.8)	135	(0.7)	158	(0.9)	154	(0.8)	
Living with mother's parents											<0.001
No	16264	(88.2)	16453	(89.2)	16559	(89.8)	16559	(89.8)	16496	(89.5)	
Yes	2174	(11.8)	1986	(10.8)	1879	(10.2)	1879	(10.2)	1942	(10.5)	
Living with partner's parents											<0.001
No	16399	(88.9)	16270	(88.2)	16267	(88.2)	16257	(88.2)	16117	(87.4)	
Yes	2039	(11.1)	2169	(11.8)	2171	(11.8)	2181	(11.8)	2321	(12.6)	
Stressful event											0.031
No	10275	(55.7)	10330	(56.0)	10455	(56.7)	10412	(56.5)	10560	(57.3)	
Yes	8163	(44.3)	8109	(44.0)	7983	(43.3)	8026	(43.5)	7878	(42.7)	
Intimate partner violence											<0.001
No	15453	(83.8)	15720	(85.3)	15900	(86.2)	16018	(86.9)	16098	(87.3)	
Yes	2986	(16.2)	2720	(14.8)	2538	(13.8)	2420	(13.1)	2340	(12.7)	
Negative attitude toward pregnancy											<0.001
No	16894	(91.6)	17002	(92.2)	17105	(92.8)	17177	(93.2)	17111	(92.8)	
Yes	1544	(8.4)	1437	(7.8)	1333	(7.2)	1261	(6.8)	1327	(7.2)	
History of depression, anxiety disorder, dysautonomia, or schizophrenia											0.522
No	16961	(92.0)	16999	(92.2)	17026	(92.3)	17015	(92.3)	16951	(91.9)	
Yes	1477	(8.0)	1440	(7.8)	1412	(7.7)	1423	(7.7)	1487	(8.1)	
Kessler Psychological Distress Scale (K6) score											<0.001
0–12	17629	(95.6)	17848	(96.8)	17886	(97.0)	17921	(97.2)	17861	(96.9)	
⩾13	809	(4.4)	591	(3.2)	552	(3.0)	517	(2.8)	578	(3.1)	

Values show the imputed data for the 92191 mothers.

### Statistical analysis

To calculate the crude and adjusted odds ratios (ORs) and their 95% CIs for each outcome, marginal structural models were fitted to a pseudo-population created using the inverse probability of treatment weighting such that no association existed between the potential confounders and the exposure variable; in other words, all backdoor paths were blocked (Cole & Hernán, 2008; Hernán & Robins, 2020; Sato & Matsuyama, 2003). The exposure variable was the quintile of omega-3 PUFA intake, and the first quintile was used as the reference. Outcome variables were each of the cases of infant maltreatment.

Loss to follow-up (1.44% at 1 month and 5.91% at 6 months) was treated using the inverse probability of censoring weighting (Hernán & Robins, 2020). Missing data were treated using multiple imputation (⩽1% for all variables excluding 2.47% for parity and 7.24% for annual household income). We created 10 imputed datasets using chained equations (van Buuren, 2007), and the results were combined using standard rules (Rubin, 2004). All analyses were performed using SAS software (version 9.4; SAS Institute Inc.) or R 4.0.0.

#### Sensitivity analysis

To assess the robustness of the results, ORs were calculated using multivariable logistic regression analysis. In addition, the analysis was repeated using energy-adjusted fish intake in place of omega-3 PUFA intake. To evaluate unmeasured confounding, the *E*-value (Ding & VanderWeele, 2016; VanderWeele & Ding, 2017), which is the minimum association required to completely cancel out the observed association, was calculated.

#### Additional analysis

To assess the assumption of positivity, the weights used for creating the pseudo-population where no association existed between the covariates and exposure as well as loss to follow-up as missing at random were summarized. Generalized variance inflation factors were calculated to assess multicollinearity.

## Results

A total of 92 191 mothers were analyzed; of these, 73.7% were less than 35 years of age, 74.0% had a pre-pregnancy body mass index of 18.5–25, and 43.4% were primiparous. Median omega-3 PUFA values, adjusted for total energy intake, for each quintile were 0.96, 1.30, 1.55, 1.82, and 2.31 g/day, respectively. Table 1 shows the participant characteristics according to omega-3 PUFA intake. Omega-3 PUFA intake was associated with almost all the potential confounders. In contrast, no apparent association was observed between omega-3 PUFA intake and the potential confounders in a pseudo-population created using the inverse probability of treatment weighting (all *p* values >0.870; online Supplementary eTable 1). Compared with mothers who were included in the analysis (*n* = 92 191), those who were excluded (*n* = 1661), due to lack of responses or missing data on omega-3 PUFA intake during pregnancy, tended, in descending order, to be younger (Cramer's *V* = 0.025), to be unmarried (Cramer's *V* = 0.021), and to have psychological distress (Cramer's *V* = 0.019).

Prevalence of ‘hitting the baby’, ‘shaking the baby very hard when he/she cries’, ‘shook the child very hard in the past month’, and ‘leaving the baby alone at home’ was 1.00, 17.79, 1.33, and 15.65%, respectively. Table 2 shows the prevalence, numbers, and crude and adjusted ORs (95% CIs) for the cases of each item according to the quintile of omega-3 PUFA intake, with the first quintile as the reference. Adjusted ORs (95% CIs) for the second through fifth quintiles were as follows: for ‘hitting the baby’, 0.93 (0.77–1.13), 0.79 (0.64–0.97), 0.78 (0.64–0.96), and 0.72 (0.59–0.89); for ‘shaking the baby very hard when he/she cries’, 0.98 (0.93–1.03), 0.94 (0.90–0.997), 0.93 (0.88–0.98), and 0.88 (0.83–0.93); for ‘shook the child very hard in the past month’, 0.87 (0.73–1.04), 0.81 (0.67–0.97), 0.73 (0.61–0.89), and 0.78 (0.65–0.94); and for ‘leaving the baby alone at home’, 0.92 (0.87–0.98), 0.91 (0.86–0.97), 0.94 (0.88–0.99), and 0.85 (0.80–0.90), respectively. A linear trend was observed for all outcomes.

**Table 2.** Odds ratios (95% CIs) for cases of infant maltreatment according to quintile for energy-adjusted omega-3 polyunsaturated fatty acid (PUFA) intake during pregnancy

	Quintile for energy-adjusted omega-3 PUFA intake during pregnancy					
	1 (low)	2	3	4	5 (high)	
	(*n* = 18 438)	(*n* = 18 439)	(*n* = 18 438)	(*n* = 18 438)	(*n* = 18 438)	*p* value for trend
**Physical abuse**						
Hitting the baby (at 1 month)						
Prevalence, %	1.22	1.10	0.92	0.88	0.85	
Cases, *n*	224	203	169	163	158	
Crude odds ratio	1.00 (Ref.)	0.91 (0.75–1.10)	0.75 (0.61–0.92)	0.72 (0.59–0.89)	0.70 (0.57–0.86)	<0.001
Adjusted^a^ odds ratio	1.00 (Ref.)	0.93 (0.77–1.13)	0.79 (0.64–0.97)	0.78 (0.64–0.96)	0.72 (0.59–0.89)	<0.001
Shaking the baby very hard when he/she cries (at 1 month)						
Prevalence, %	19.3	18.4	17.6	17.1	16.6	
Cases, *n*	3566	3385	3242	3153	3055	
Crude odds ratio	1.00 (Ref.)	0.94 (0.89–0.99)	0.89 (0.84–0.94)	0.86 (0.82–0.91)	0.83 (0.78–0.87)	<0.001
Adjusted^a^ odds ratio	1.00 (Ref.)	0.98 (0.93–1.03)	0.94 (0.90–1.00)	0.93 (0.88–0.98)	0.88 (0.83–0.93)	<0.001
Shook the child very hard in the past month (at 6 months)						
Prevalence, %	1.69	1.38	1.22	1.08	1.18	
Cases, *n*	313	255	225	198	218	
Crude odds ratio	1.00 (Ref.)	0.81 (0.68–0.97)	0.72 (0.60–0.86)	0.63 (0.52–0.76)	0.69 (0.58–0.83)	<0.001
Adjusted^a^ odds ratio	1.00 (Ref.)	0.87 (0.73–1.04)	0.81 (0.67–0.97)	0.73 (0.61–0.89)	0.78 (0.65–0.94)	0.002
**Neglect**						
Leaving the baby alone at home (at 1 month)						
Prevalence, %	16.7	15.7	15.5	15.8	14.5	
Cases, *n*	3078	2887	2866	2919	2666	
Crude odds ratio	1.00 (Ref.)	0.93 (0.88–0.98)	0.92 (0.87–0.97)	0.94 (0.89–0.99)	0.84 (0.80–0.89)	<0.001
Adjusted^a^ odds ratio	1.00 (Ref.)	0.92 (0.87–0.98)	0.91 (0.86–0.97)	0.94 (0.88–0.99)	0.85 (0.80–0.90)	<0.001

a Adjusted for maternal age, pre-pregnancy body mass index, highest education level, full-time work, annual household income, smoking status, alcohol intake, parity, marital status, living with mother's parents, living with partner's parents, stressful events, intimate partner violence, negative attitude toward pregnancy, history of depression, anxiety disorder, dysautonomia, or schizophrenia, and psychological distress.

No meaningful differences were observed in the results derived using multivariable logistic regression models and those derived from the main analysis (online Supplementary eTable 2). Online Supplementary eTable 3 shows the results for fish intake. A weak but similar tendency was observed relative to that for omega-3 PUFA intake. The *E*-values corresponding to the fifth quintile for ‘hitting the baby’ (OR 0.72) and the fourth quintile for ‘shook the child very hard’ (OR 0.73) were 2.11 and 2.07, respectively. These values suggest that relatively strong unmeasured potential confounders would be necessary to cancel out the observed association.

The mean value (SD) of standardized treatment weights for the omega-3 PUFA model was 1.000 (0.114). The mean values (SDs) of censoring weights at 1 and 6 months were 1.015 (0.011) and 1.063 (0.050), respectively. Online Supplementary eTable 4 shows the details and weights for the fish model. The findings suggest that a positivity violation was unlikely to occur. Multicollinearity was not detected among the covariates; that is, all generalized variance inflation factors were below 1.34.

## Discussion

Our analyses revealed that even when controlling for up to 16 carefully selected potential confounders, omega-3 PUFA intake during pregnancy was associated with fewer cases of hitting the baby, shaking the baby very hard at 1 and 6 months, and leaving the baby alone at home. These relationships were more salient for omega-3 PUFA intake than for fish intake. Importantly, clear dose-response relationships were observed in most cases. These findings support our hypothesis that mothers with a higher intake of omega-3 PUFAs during pregnancy exhibit less infant maltreatment.

Our findings on infant abuse are consistent with those of a meta-analysis (Gajos & Beaver, 2016) of 40 studies, including both randomized controlled trials and cohort studies, that examined 73 effect sizes and concluded that omega-3 PUFA intake reduces violent and aggressive behaviors. Hitting and/or shaking babies constitutes physical abuse among the types of infant maltreatment. The association was stronger for omega-3 PUFA intake than for fish consumption, which might be due to the fact that not all species of fish contain high levels of omega-3 PUFAs. In addition, our findings showed a relatively stronger association and clearer dose-dependent response, which was likely due to our participants being perinatal mothers who recently experienced pregnancy, delivery, and nurturing, which may be regarded as a series of stressful events (Holmes & Rahe, 1967), despite the desire of these women to have a baby. Previous studies have pointed out that the effects of omega-3 PUFAs may be augmented in stressful and/or vulnerable situations (Appleton et al., 2008; Hamazaki & Hamazaki, 2008). To our knowledge, this is the first study to suggest that the suppressing effect of omega-3 PUFAs on violent and aggressive behaviors might also be applicable to child abuse.

The present findings on neglect are also consistent with evidence from animal studies demonstrating that dams deficient in omega-3 PUFAs exhibit reduced maternal nurturing behavior, such as less licking and grooming and poorer nesting behaviors (Asch et al., 2019; Harauma et al., 2016). Notably, approximately 40% of dams with deficits in omega-3 PUFAs attacked or neglected pups on the day of delivery, with some pups from omega-3 PUFA-deficient dams dying (Harauma et al., 2016). In humans, it is known that more infants die of neglect than of physical abuse (Ministry of Health, Labour and Welfare, 2020; Palusci & Covington, 2014), suggesting that maternal nurturing behavior plays a pivotal role in the survival of offspring. To our knowledge, this is also the first study to suggest that the beneficial effects of omega-3 PUFAs might also be applicable to human neglecting behavior.

Although the precise mechanisms underlying the impact of omega-3 PUFA intake on infant maltreatment remain unclear, several possible pathways exist. The first is via stress reduction. Omega-3 PUFAs modulate a wide range of neural substrates that contribute to emotional regulation, such as noradrenaline, dopamine, serotonin, and endocannabinoid systems (Freeman et al., 2006; Lafourcade et al., 2011). In addition, omega-3 PUFAs reduce stressor-evoked augmentation of autonomic activity (Ginty & Conklin, 2012; Hamazaki et al., 2005; Matsumura et al., 2017) as a whole-body preparation for fight-or-flight stress responses. Thus, intake of omega-3 PUFAs may reduce infant maltreatment by reducing the mother's behavioral stress response toward the baby, hitting and shaking as a fight response, and escaping from the baby (leaving the baby alone at home) as a flight response.

The second pathway is via a reduction in depression. Omega-3 PUFAs are known to exert antidepressant effects (Freeman et al., 2006; Lin & Su, 2007), including in mothers with postpartum depression (Urech et al., 2020; Zhang et al., 2020). Because postpartum depression is a risk factor for infant maltreatment (Stith et al., 2009), intake of omega-3 PUFAs may reduce infant maltreatment via a reduction in postpartum depression.

The third pathway is via behavioral changes in infants. Omega-3 PUFA supplementation in children is effective for reducing externalizing behaviors such as aggression, non-compliance, and hyperactivity (Portnoy, Raine, Liu, & Hibbeln, 2018). Given that maternal concentrations of essential fatty acids, including omega-3 PUFAs, are correlated with those in newborn babies (Al, Hornstra, van der Schouw, Bulstra-Ramakers, & Huisjes, 1990), omega-3 PUFAs in infants that were transferred from mothers may play a role in reducing fretful behavior in infants. Triggers of physical abuse-related deaths include crying and disobedience (Palusci & Covington, 2014), which might be reduced by the intake of omega-3 PUFAs.

There are many identified risk factors for child maltreatment. However, many risk factors are hard to address in intervention, such as unplanned pregnancy, unemployment, psychopathology, drug abuse, and lower age (Clement et al., 2016; Palusci, 2011; Stith et al., 2009; Wu et al., 2004). It would be easier to increase omega-3 PUFA intake, however. Conveniently, omega-3 PUFAs seem to effectively reduce anger/hyper-reactivity, depression, and stress in mothers, which a meta-analysis identified as strong risk factors (Stith et al., 2009). Moreover, pregnant women are usually deficient in omega-3 PUFAs (Hornstra, Al, van Houwelingen, & Foreman-van Drongelen, 1995; Markhus et al., 2015) and are therefore recommended to increase their intake during pregnancy (Coletta, Bell, & Roman, 2010). This means that it is not necessary to establish completely new systems for recommending increased omega-3 PUFA intake, but rather to emphasize the importance of omega-3 PUFA intake in ongoing nutritional guidance for pregnant women. One efficient method for ensuring sufficient intake of omega-3 PUFAs includes eating blue-backed omega-3 PUFA-rich fish, such as sardines, saury, and horse mackerel. Conveniently, blue-backed fishes are small and are not at the top of the food chain, so biological concentrations of toxic chemicals such as mercury and/or PCBs are rare. Flaxseed oil is rich in the omega-3 PUFA *α*-linolenic acid; however, *α*-linolenic acid needs to be converted into EPA and subsequently DHA before use, and so is less widely recommended. It usually takes 6–8 weeks before the effects of omega-3 PUFA supplementation manifest (Gajos & Beaver, 2016; Zhang et al., 2020), and therefore commencing this dietary practice as early as possible is recommended. Other recommended fishes may be found elsewhere in the literature (Coletta et al., 2010; Mozaffarian & Rimm, 2006) and in local guidelines.

This study has several strengths. First, our sample size was large, including over 90 000 mothers, which resulted in successful detection of differences in physical abuse with a low prevalence of approximately 1%. Second, the participants were enrolled from multiple regions throughout Japan from 2011 to 2014 and are therefore reflective of the entire nation. Third, the dropout rate was relatively low (approximately 5.9% at 6 months postpartum), suggesting the existence of low selection bias due to dropout. Fourth, statistical models used in the study were conditioned on a wide range of potential confounders, thereby possibly yielding estimates close to the true effects. Fifth, missing value rates for maltreatment items excluding dropout were low (maximum of 0.70%) compared with those for other sensitive items related to income (7.24%), demonstrating that participants answered these items without being too defensive or having a psychological set. Finally, our findings may be generalizable to other populations given that the assumed mechanisms are biological.

This study has several limitations. First, we examined infant maltreatment only in mothers. Mothers are the leading offenders of infant maltreatment deaths in Japan (Ministry of Health, Labour and Welfare, 2020), but further research examining this association in fathers and non-parent caregivers is needed (Stith et al., 2009). Second, infant maltreatment was measured using a self-reported questionnaire. Although a gold standard measure for infant maltreatment is lacking (Moody et al., 2018), further studies using objective markers such as maladaptive responses in the hypothalamic-pituitary-adrenal axis and DNA methylation in children (McGowan et al., 2009; Weaver et al., 2004) would be beneficial. Third, we did not measure sexual abuse, partly because we were unable to determine a hypothetical link between omega-3 PUFA intake and sexual abuse. Although infant death due to sexual abuse is rare yet not non-existent (Palusci & Covington, 2014), further studies pursuing this issue are necessary. Fourth, we measured child maltreatment only until 6 months postpartum. Continuous follow-up assessment is necessary for at least a decade to cover the entire period in which potential maltreatment of children could occur. Fifth, we included various potential confounders in the model but cannot rule out the possible existence of unmeasured potential confounders. Thus, randomized controlled trials are warranted to establish a standard prevention strategy against maltreatment. Finally, we measured omega-3 PUFA intake using an FFQ only. The FFQ has been validated for use in large-scale Japanese epidemiologic studies (Sasaki et al., 2003; Yokoyama et al., 2016), but it has not been validated for use with pregnant women. In general, the FFQ is less accurate than the food weighing method. Therefore, further research measuring omega-3 PUFA intake using the food weighing method and/or by directly measuring the composition of erythrocyte PUFAs should be conducted.

## Conclusions

This study demonstrated that higher maternal intake of omega-3 PUFAs was associated with fewer cases of hitting, hard shaking, and leaving babies alone at home, suggesting a lower risk of infant maltreatment. Our results indicate the potential applicability of omega-3 PUFAs in reducing infant maltreatment.

## Supplementary material

For supplementary material accompanying this paper visit https://doi.org/10.1017/S0033291721002427. click here to view supplementary material

## Appendix

Members of the JECS Group as of 2021: Michihiro Kamijima (Principal Investigator, Nagoya City University, Nagoya, Japan), Shin Yamazaki (National Institute for Environmental Studies, Tsukuba, Japan), Yukihiro Ohya (National Center for Child Health and Development, Tokyo, Japan), Reiko Kishi (Hokkaido University, Sapporo, Japan), Nobuo Yaegashi (Tohoku University, Sendai, Japan), Koichi Hashimoto (Fukushima Medical University, Fukushima, Japan), Chisato Mori (Chiba University, Chiba, Japan), Shuichi Ito (Yokohama City University, Yokohama, Japan), Zentaro Yamagata (University of Yamanashi, Chuo, Japan), Hidekuni Inadera (University of Toyama, Toyama, Japan), Takeo Nakayama (Kyoto University, Kyoto, Japan), Hiroyasu Iso (Osaka University, Suita, Japan), Masayuki Shima (Hyogo College of Medicine, Nishinomiya, Japan), Youichi Kurozawa (Tottori University, Yonago, Japan), Narufumi Suganuma (Kochi University, Nankoku, Japan), Koichi Kusuhara (University of Occupational and Environmental Health, Kitakyushu, Japan), and Takahiko Katoh (Kumamoto University, Kumamoto, Japan).
