# Supplementary material for: Omega-3 fatty acid intake during pregnancy and risk of infant maltreatment: a nationwide birth cohort – the Japan Environment and Children's Study
Source: Psychol Med. 2021 Jun 25;53(3):995–1004. doi: 10.1017/S0033291721002427 (PMC9975990; doi:10.1017/S0033291721002427)
Supplement: Supplementary file 1 [file S0033291721002427sup001.docx]

Supplement for

**Omega-3 Fatty Acid Intake During Pregnancy and Risk of Infant Maltreatment: A Nationwide Birth Cohort—The Japan Environment and Children’s Study**

Kenta Matsumura, PhD; Kei Hamazaki, MD, PhD; Akiko Tsuchida, PhD; Hidekuni Inadera, MD, PhD; and the Japan Environment and Children’s Study (JECS) Group

**Contents**

eTable 1. Characteristics of pseudo-population created using the inverse probability of treatment weighting according to quintile for energy-adjusted omega-3 polyunsaturated fatty acid (PUFA) intake during pregnancy.

eTable 2. Odds ratios (95% CIs) for cases of each type of infant maltreatment according to quintile for energy-adjusted omega-3 polyunsaturated fatty acid (PUFA) intake during pregnancy, derived from multivariable logistic regression analysis.

eTable 3. Odds ratios (95% CIs) for cases of each type of infant maltreatment according to quintile for energy-adjusted fish intake during pregnancy.

eTable 4. Summary of the inverse probability weights used for each marginal structural model.

**eTable 1. Characteristics of pseudo-population created using the inverse probability of treatment weighting according to quintile for energy-adjusted omega-3 polyunsaturated fatty acid (PUFA) intake during pregnancy.**

| Variable | | **Quintile for energy-adjusted omega-3 PUFA intake during pregnancy** | | | | | | | | | | | | | |  | |  | | |  | | |  |
| --- | --- | --- | --- | --- | --- | --- | --- | --- | --- | --- | --- | --- | --- | --- | --- | --- | --- | --- | --- | --- | --- | --- | --- | --- |
|  |  | **1 (low)** |  |  | **2** |  |  | **3** |  | |  | **4** | |  | |  | **5 (high)** | | |  | | |  | |
|  |  | **(n = 18,433)** | |  | **(n = 18,439)** | |  | **(n = 18,439)** | | |  | **(n = 18,440)** | | | |  | **(n = 18,438)** | | | | | |  | |
|  |  | **n** | **(%)** |  | **n** | **(%)** |  | **n** | **(%)** | |  | **n** | | **(%)** | |  | **n** | | | **(%)** | | | ***p*** | |
| Median intake, g/day | | 0.96 |  |  | 1.30 |  |  | 1.55 |  |  | 1.82 | |  | |  | 2.31 | | |  | | |  | | |
| Maternal age, y | |  |  |  |  |  |  |  |  |  |  | |  | |  |  | | |  | | | 1.000 | | |
|  | <25 | 1,951 | (10.6) |  | 1,940 | (10.5) |  | 1,942 | (10.5) | |  | 1,943 | | (10.5) | |  | 1,939 | | | (10.5) | | |  | |
|  | 25 to <30 | 5,215 | (28.3) |  | 5,221 | (28.3) |  | 5,219 | (28.3) | |  | 5,224 | | (28.3) | |  | 5,210 | | | (28.3) | | |  | |
|  | 30 to <35 | 6,473 | (35.1) |  | 6,477 | (35.1) |  | 6,472 | (35.1) | |  | 6,479 | | (35.1) | |  | 6,482 | | | (35.2) | | |  | |
|  | ≥35 | 4,794 | (26.0) |  | 4,801 | (26.0) |  | 4,807 | (26.1) | |  | 4,795 | | (26.0) | |  | 4,807 | | | (26.1) | | |  | |
| Pre-pregnancy body mass index, kg/m^2^ | |  |  |  |  |  |  |  |  |  |  | |  | |  |  | | |  | | | 1.000 | | |
|  | <18.5 | 2,987 | (16.2) |  | 2,989 | (16.2) |  | 2,987 | (16.2) | |  | 2,982 | | (16.2) | |  | 2,992 | | | (16.2) | | |  | |
|  | 18.5 to <25 | 13,495 | (73.2) |  | 13,509 | (73.3) |  | 13,505 | (73.2) | |  | 13,519 | | (73.3) | |  | 13,503 | | | (73.2) | | |  | |
|  | ≥25 | 1,951 | (10.6) |  | 1,941 | (10.5) |  | 1,947 | (10.6) | |  | 1,939 | | (10.5) | |  | 1,942 | | | (10.5) | | |  | |
| Highest education level, y | |  |  |  |  |  |  |  |  |  |  | |  | |  |  | | |  | | | 1.000 | | |
|  | ≤12 | 6,686 | (36.3) |  | 6,677 | (36.2) |  | 6,677 | (36.2) | |  | 6,684 | | (36.3) | |  | 6,674 | | | (36.2) | | |  | |
|  | >12 to <16 | 7,747 | (42.0) |  | 7,754 | (42.1) |  | 7,752 | (42.0) | |  | 7,755 | | (42.1) | |  | 7,751 | | | (42.0) | | |  | |
|  | ≥16 | 4,000 | (21.7) |  | 4,008 | (21.7) |  | 4,010 | (21.8) | |  | 4,001 | | (21.7) | |  | 4,012 | | | (21.8) | | |  | |
| Full-time work | |  |  |  |  |  |  |  |  |  |  | |  | |  |  | | |  | | | 1.000 | | |
|  | No | 12,772 | (69.3) |  | 12,761 | (69.2) |  | 12,761 | (69.2) | |  | 12,764 | | (69.2) | |  | 12,770 | | | (69.3) | | |  | |
|  | Yes | 5,660 | (30.7) |  | 5,678 | (30.8) |  | 5,678 | (30.8) | |  | 5,677 | | (30.8) | |  | 5,668 | | | (30.7) | | |  | |
| Annual household income, million JPY | | |  |  |  |  |  |  |  | |  |  | |  | |  |  | | |  | | | 1.000 | |
|  | <4 | 7,497 | (40.7) |  | 7,491 | (40.6) |  | 7,486 | (40.6) | |  | 7,487 | | (40.6) | |  | 7,494 | | | (40.6) | | |  | |
|  | 4 to <6 | 6,033 | (32.7) |  | 6,058 | (32.9) |  | 6,058 | (32.9) | |  | 6,053 | | (32.8) | |  | 6,053 | | | (32.8) | | |  | |
|  | ≥6 | 4,903 | (26.6) |  | 4,890 | (26.5) |  | 4,895 | (26.6) | |  | 4,900 | | (26.6) | |  | 4,892 | | | (26.5) | | |  | |
| Smoking status | |  |  |  |  |  |  |  |  |  |  | |  | |  |  | | |  | | | 1.000 | | |
|  | Never | 10,641 | (57.7) |  | 10,654 | (57.8) |  | 10,647 | (57.7) | |  | 10,653 | | (57.8) | |  | 10,652 | | | (57.8) | | |  | |
|  | Former | 6,945 | (37.7) |  | 6,942 | (37.7) |  | 6,951 | (37.7) | |  | 6,943 | | (37.7) | |  | 6,941 | | | (37.6) | | |  | |
|  | Current | 847 | (4.6) |  | 842 | (4.6) |  | 842 | (4.6) | |  | 845 | | (4.6) | |  | 845 | | | (4.6) | | |  | |
| Alcohol intake | |  |  |  |  |  |  |  |  |  |  | |  | |  |  | | |  | | | 1.000 | | |
|  | Never | 6,151 | (33.4) |  | 6,166 | (33.4) |  | 6,166 | (33.4) | |  | 6,166 | | (33.4) | |  | 6,166 | | | (33.4) | | |  | |
|  | Former | 11,771 | (63.9) |  | 11,763 | (63.8) |  | 11,763 | (63.8) | |  | 11,763 | | (63.8) | |  | 11,766 | | | (63.8) | | |  | |
|  | Current | 511 | (2.8) |  | 510 | (2.8) |  | 510 | (2.8) | |  | 512 | | (2.8) | |  | 507 | | | (2.8) | | |  | |
| Parity | |  |  |  |  |  |  |  |  |  |  | |  | |  |  | | |  | | | 0.998 | | |
|  | Primipara | 7,925 | (43.0) |  | 7,916 | (42.9) |  | 7,924 | (43.0) | |  | 7,927 | | (43.0) | |  | 7,898 | | | (42.8) | | |  | |
|  | Multipara | 10,508 | (57.0) |  | 10,522 | (57.1) |  | 10,515 | (57.0) | |  | 10,513 | | (57.0) | |  | 10,540 | | | (57.2) | | |  | |
| Marital status | |  |  |  |  |  |  |  |  |  |  | |  | |  |  | | |  | | | 1.000 | | |
|  | Married | 17,574 | (95.3) |  | 17,576 | (95.3) |  | 17,576 | (95.3) | |  | 17,580 | | (95.3) | |  | 17,575 | | | (95.3) | | |  | |
|  | Single | 691 | (3.8) |  | 693 | (3.8) |  | 695 | (3.8) | |  | 691 | | (3.8) | |  | 695 | | | (3.8) | | |  | |
|  | Divorced or widowed | 168 | (0.9) |  | 170 | (0.9) |  | 168 | (0.9) | |  | 170 | | (0.9) | |  | 168 | | | (0.9) | | |  | |
| Living with mother's parents | |  |  |  |  |  |  |  |  |  |  | |  | |  |  | | |  | | | 0.999 | | |
|  | No | 16,450 | (89.2) |  | 16,465 | (89.3) |  | 16,463 | (89.3) | |  | 16,470 | | (89.3) | |  | 16,454 | | | (89.2) | | |  | |
|  | Yes | 1,983 | (10.8) |  | 1,974 | (10.7) |  | 1,976 | (10.7) | |  | 1,970 | | (10.7) | |  | 1,984 | | | (10.8) | | |  | |
| Living with partner's parents | |  |  |  |  |  |  |  |  |  |  | |  | |  |  | | |  | | | 1.000 | | |
|  | No | 16,268 | (88.3) |  | 16,264 | (88.2) |  | 16,265 | (88.2) | |  | 16,270 | | (88.2) | |  | 16,260 | | | (88.2) | | |  | |
|  | Yes | 2,165 | (11.7) |  | 2,174 | (11.8) |  | 2,174 | (11.8) | |  | 2,170 | | (11.8) | |  | 2,178 | | | (11.8) | | |  | |
| Stressful event | |  |  |  |  |  |  |  |  |  |  | |  | |  |  | | |  | | | 1.000 | | |
|  | No | 10,398 | (56.4) |  | 10,408 | (56.5) |  | 10,405 | (56.4) | |  | 10,407 | | (56.4) | |  | 10,408 | | | (56.5) | | |  | |
|  | Yes | 8,035 | (43.6) |  | 8,031 | (43.6) |  | 8,034 | (43.6) | |  | 8,033 | | (43.6) | |  | 8,030 | | | (43.6) | | |  | |
| Intimate partner violence | |  |  |  |  |  |  |  |  |  |  | |  | |  |  | | |  | | | 0.870 | | |
|  | No | 15,801 | (85.7) |  | 15,834 | (85.9) |  | 15,819 | (85.8) | |  | 15,844 | | (85.9) | |  | 15,875 | | | (86.1) | | |  | |
|  | Yes | 2,632 | (14.3) |  | 2,605 | (14.1) |  | 2,620 | (14.2) | |  | 2,596 | | (14.1) | |  | 2,563 | | | (13.9) | | |  | |
| Negative attitude towards pregnancy | | |  |  |  |  |  |  |  | |  |  | |  | |  |  | | |  | | | 1.000 | |
|  | No | 17,049 | (92.5) |  | 17,057 | (92.5) |  | 17,060 | (92.5) | |  | 17,060 | | (92.5) | |  | 17,056 | | | (92.5) | | |  | |
|  | Yes | 1,384 | (7.5) |  | 1,381 | (7.5) |  | 1,379 | (7.5) | |  | 1,380 | | (7.5) | |  | 1,382 | | | (7.5) | | |  | |
| History of depression, anxiety disorder, dysautonomia, or schizophrenia | | | | | |  |  |  |  | |  |  | |  | |  |  | | |  | | | 0.999 | |
|  | No | 16,979 | (92.1) |  | 16,994 | (92.2) |  | 16,991 | (92.2) | |  | 16,986 | | (92.1) | |  | 16,995 | | | (92.2) | | |  | |
|  | Yes | 1,454 | (7.9) |  | 1,444 | (7.8) |  | 1,448 | (7.9) | |  | 1,455 | | (7.9) | |  | 1,443 | | | (7.8) | | |  | |
| Kessler Psychological Distress Scale (K6) score | | | |  |  |  |  |  |  | |  |  | |  | |  |  | | |  | | | 1.000 | |
|  | 0-12 | 17,818 | (96.7) |  | 17,829 | (96.7) |  | 17,829 | (96.7) | |  | 17,831 | | (96.7) | |  | 17,829 | | | (96.7) | | |  | |
|  | ≥13 | 615 | (3.3) |  | 610 | (3.3) |  | 610 | (3.3) | |  | 609 | | (3.3) | |  | 610 | | | (3.3) | | |  | |

Values show the imputed data for the 92,191 mothers.

**eTable 2. Odds ratios (95% CIs) for cases of each type of infant maltreatment according to quintile for energy-adjusted omega-3 polyunsaturated fatty acid (PUFA) intake during pregnancy, derived from multivariable logistic regression analysis.**

|  |  |  | **Quintile for energy-adjusted omega 3 PUFA intake during pregnancy** | | | |  |  |
| --- | --- | --- | --- | --- | --- | --- | --- | --- |
|  |  |  | **1 (low)** | **2** | **3** | **4** | **5 (high)** | ***p*-value** |
|  |  |  | **(n = 18,438)** | **(n = 18,439)** | **(n = 18,438)** | **(n = 18,438)** | **(n = 18,438)** | **for trend** |
| Physical abuse | | |  |  |  |  |  |  |
|  | Hitting the baby (at 1 month) | | |  |  |  |  |  |
|  |  | Prevalence, % | 1.22 | 1.10 | 0.92 | 0.89 | 0.86 |  |
|  |  | Cases, n | 225 | 203 | 170 | 164 | 159 |  |
|  |  | Crude odds ratio | 1.00 (Ref.) | 0.90 (0.74, 1.09) | 0.75 (0.62, 0.92) | 0.73 (0.59, 0.89) | 0.71 (0.57, 0.87) | < 0.001 |
|  |  | Adjusted^a^ odds ratio | 1.00 (Ref.) | 0.94 (0.77, 1.14) | 0.80 (0.65, 0.98) | 0.78 (0.63, 0.96) | 0.74 (0.60, 0.91) | < 0.001 |
|  | Shaking the baby very hard when he/she cries (at 1 month) | | | |  |  |  |  |
|  |  | Prevalence, % | 19.3 | 18.4 | 17.6 | 17.1 | 16.6 |  |
|  |  | Cases, n | 3,564 | 3,387 | 3,244 | 3,155 | 3,053 |  |
|  |  | Crude odds ratio | 1.00 (Ref.) | 0.94 (0.89, 0.99) | 0.89 (0.84, 0.94) | 0.86 (0.82, 0.91) | 0.83 (0.78, 0.87) | < 0.001 |
|  |  | Adjusted^a^ odds ratio | 1.00 (Ref.) | 0.98 (0.93, 1.04) | 0.95 (0.90, 1.00) | 0.93 (0.88, 0.98) | 0.87 (0.83, 0.92) | < 0.001 |
|  | Shook the child very hard in the past month (at 6 months) | | | | | |  |  |
|  |  | Prevalence, % | 1.71 | 1.41 | 1.22 | 1.09 | 1.20 |  |
|  |  | Cases, n | 315 | 261 | 226 | 201 | 222 |  |
|  |  | Crude odds ratio | 1.00 (Ref.) | 0.83 (0.70, 0.98) | 0.71 (0.60, 0.86) | 0.64 (0.53, 0.76) | 0.70 (0.59, 0.84) | < 0.001 |
|  |  | Adjusted^a^ odds ratio | 1.00 (Ref.) | 0.88 (0.74, 1.05) | 0.80 (0.66, 0.95) | 0.72 (0.60, 0.86) | 0.77 (0.65, 0.92) | < 0.001 |
| Neglect | |  |  |  |  |  |  |  |
|  | Leaving the baby alone at home (at 1 month) | | | |  |  |  |  |
|  |  | Prevalence, % | 16.7 | 15.7 | 15.6 | 15.8 | 14.5 |  |
|  |  | Cases, n | 3,082 | 2,889 | 2,869 | 2,918 | 2,668 |  |
|  |  | Crude odds ratio | 1.00 (Ref.) | 0.93 (0.88, 0.98) | 0.92 (0.87, 0.97) | 0.94 (0.89, 0.99) | 0.84 (0.80, 0.89) | < 0.001 |
|  |  | Adjusted^a^ odds ratio | 1.00 (Ref.) | 0.92 (0.87, 0.97) | 0.91 (0.86, 0.97) | 0.93 (0.88, 0.99) | 0.85 (0.80, 0.90) | < 0.001 |

^a^Adjusted for maternal age, pre-pregnancy body mass index, highest education level, full-time work, annual household income, smoking status, alcohol intake, parity, marital status, living with mother’s parents, living with partner’s parents, stressful events, intimate partner violence, negative attitude toward pregnancy, history of depression, anxiety disorder, dysautonomia, or schizophrenia, and psychological distress.

**eTable 3. Odds ratios (95% CIs) for cases of each type of infant maltreatment according to quintile for energy-adjusted fish intake during pregnancy.**

|  |  |  | **Quintile for energy-adjusted fish intake during pregnancy** | | | | | |  | |  | |
| --- | --- | --- | --- | --- | --- | --- | --- | --- | --- | --- | --- | --- |
|  |  |  | **1 (low)** | **2** | | **3** | | **4** | | **5 (high)** | | ***p*-value** |
|  |  |  | **(n = 18,438)** | **(n = 18,439)** | | **(n = 18,438)** | | **(n = 18,438)** | | **(n = 18,438)** | | **for trend** |
| Physical abuse | | |  |  | |  | |  | |  | |  |
|  | Hitting the baby (at 1 month) | | |  | |  | |  | |  | |  |
|  |  | Prevalence, % | 1.17 | 0.96 | | 1.00 | | 0.94 | | 0.90 | |  |
|  |  | Cases, n | 216 | 177 | | 185 | | 173 | | 165 | |  |
|  |  | Crude odds ratio | 1.00 (Ref.) | 0.82 (0.67, 1.00) | | 0.86 (0.70, 1.05) | | 0.80 (0.65, 0.98) | | 0.76 (0.62, 0.94) | | 0.016 |
|  |  | Adjusted^a^ odds ratio | 1.00 (Ref.) | 0.86 (0.70, 1.06) | | 0.92 (0.75, 1.13) | | 0.88 (0.71, 1.08) | | 0.81 (0.66, 1.00) | | 0.099 |
|  | Shaking the baby very hard when he/she cries (at 1 month) | | | |  | |  | |  | |  | |
|  |  | Prevalence, % | 19.8 | 18.1 | | 17.7 | | 16.9 | | 16.5 | |  |
|  |  | Cases, n | 3,659 | 3,333 | | 3,259 | | 3,115 | | 3,035 | |  |
|  |  | Crude odds ratio | 1.00 (Ref.) | 0.89 (0.85, 0.94) | | 0.87 (0.82, 0.91) | | 0.82 (0.78, 0.87) | | 0.80 (0.75, 0.84) | | < 0.001 |
|  |  | Adjusted^a^ odds ratio | 1.00 (Ref.) | 0.97 (0.92, 1.03) | | 0.97 (0.92, 1.03) | | 0.93 (0.88, 0.99) | | 0.89 (0.84, 0.94) | | < 0.001 |
|  | Shook the child very hard in the past month (at 6 months) | | | | | | | |  | |  | |
|  |  | Prevalence, % | 1.60 | 1.40 | | 1.28 | | 1.13 | | 1.14 | |  |
|  |  | Cases, n | 295 | 258 | | 236 | | 208 | | 211 | |  |
|  |  | Crude odds ratio | 1.00 (Ref.) | 0.87 (0.73, 1.04) | | 0.80 (0.67, 0.96) | | 0.70 (0.58, 0.85) | | 0.71 (0.59, 0.86) | | < 0.001 |
|  |  | Adjusted^a^ odds ratio | 1.00 (Ref.) | 1.01 (0.85, 1.21) | | 0.96 (0.80, 1.16) | | 0.89 (0.73, 1.08) | | 0.86 (0.71, 1.04) | | 0.049 |
| Neglect | |  |  |  | |  | |  | |  | |  |
|  | Leaving the baby alone at home (at 1 month) | | | |  | |  | |  | |  | |
|  |  | Prevalence, % | 15.8 | 15.7 | | 16.1 | | 15.2 | | 15.3 | |  |
|  |  | Cases, n | 2,911 | 2,899 | | 2,976 | | 2,806 | | 2,824 | |  |
|  |  | Crude odds ratio | 1.00 (Ref.) | 0.99 (0.94, 1.05) | | 1.03 (0.97, 1.09) | | 0.96 (0.90, 1.01) | | 0.96 (0.91, 1.02) | | 0.093 |
|  |  | Adjusted^a^ odds ratio | 1.00 (Ref.) | 0.98 (0.92, 1.04) | | 1.00 (0.95, 1.06) | | 0.94 (0.88, 0.99) | | 0.95 (0.90, 1.01) | | 0.041 |

^a^Adjusted for maternal age, pre-pregnancy body mass index, highest education level, full-time work, annual household income, smoking status, alcohol intake, parity, marital status, living with mother’s parents, living with partner’s parents, stressful events, intimate partner violence, negative attitude toward pregnancy, history of depression, anxiety disorder, dysautonomia, or schizophrenia, and psychological distress.

**eTable 4. Summary of the inverse probability weights used for each marginal structural model.**

|  |  |  | |  | | **Percentile** | | | | | | | |
| --- | --- | --- | --- | --- | --- | --- | --- | --- | --- | --- | --- | --- | --- |
|  |  | **M** | | **(SD)** | | **0%** | | **1%** | | **99%** | | **100%** | |
| Omega-3 polyunsaturated fatty acid intake | | |  | |  | |  | |  | |  | |  |
|  | Standardized treatment weights | 1.000 | | (0.114) | | 0.434 | | 0.722 | | 1.359 | | 1.934 | |
|  | Censoring weights for 1 month | 1.015 | | (0.011) | | 1.003 | | 1.005 | | 1.061 | | 1.325 | |
|  | Censoring weights for 6 months | 1.063 | | (0.050) | | 1.016 | | 1.019 | | 1.261 | | 2.550 | |
| Fish intake | |  | |  | |  | |  | |  | |  | |
|  | Standardized treatment weights | 1.000 | | (0.154) | | 0.383 | | 0.623 | | 1.528 | | 2.411 | |
|  | Censoring weights for 1 month | 1.015 | | (0.011) | | 1.003 | | 1.005 | | 1.062 | | 1.327 | |
|  | Censoring weights for 6 months | 1.063 | | (0.050) | | 1.016 | | 1.020 | | 1.262 | | 2.674 | |
